# Barriers and Facilitators Related to Undertaking Physical Activities in Colorectal Cancer Patients: A Scoping Review

**DOI:** 10.3389/or.2024.1360480

**Published:** 2024-09-06

**Authors:** Hu Yan, Chang Shuying, Li Yuege, Kong Han

**Affiliations:** ^1^ School of Nursing and Health, Zhengzhou University, Zhengzhou, China; ^2^ Henan Provincial People’s Hospital, Zhengzhou, China; ^3^ School of Nursing and Health, Henan University, Kaifeng, China

**Keywords:** physical activities, colorectal cancer, barriers, facilitators, scoping review

## Abstract

**Background:**

Colorectal cancer (CRC) and its treatments cause significant acute, chronic, or latent adverse effects, leading to decreased physical function and quality of life. Robust evidence supports the positive effects of physical activity (PA) on various health outcomes in CRC patients. However, there is limited understanding regarding the factors that influence PA engagement, including facilitators, preferences, and barriers in this population.

**Purpose:**

This scoping review aims to document the breadth and depth of literature concerning the various aspects of PA participation among patients with CRC. We conducted a scoping review of PA among CRC patients.

**Methods:**

We searched several databases, including PubMed, Web of Science, Embase, and Cochrane, from their inception to 25 July 2023. Multiple reviewers were involved in all screening and data abstractions. The search yielded 834 individual citations after removing duplicates. After screening the titles and abstracts, 20 articles underwent full-text review, and 11 were included.

**Results:**

Our research findings indicate that among CRC patients, the most prevalent facilitators/preferences for PA are understanding its importance and perceiving its benefits, whereas treatment-related effects and lack of time are the most common barriers.

**Conclusion:**

CRC patients have unique facilitators and barriers concerning PA. Further research and clinical interventions are required to support and encourage this population to participate in and maintain regular PA.

## Introduction

Colorectal cancer (CRC) is the third most prevalent cancer globally and ranks the second leading cause of cancer-related mortality [1, 2]. Most colorectal cancer patients fail to participate in adequate physical activity (PA) [3–8]. Modification section: Research shows PA has more benefits for CRC patients than other cancer types such as breast cancer, including enhancements in physical fitness and quality of life [9, 10]. Physical exercise is crucial for reducing complications [11–13], CRC recurrence rates [14–17], cancer-specific mortality, and overall mortality [15, 18, 19]. However, evidence suggests that approximately 75% of CRC survivors have insufficient physical activity [6]. Research also shows a significant decline in PA levels among cancer survivors during treatment, and these levels often fail to fully recover to the pre-diagnosis level [20]. Based on this evidence, it is important to identify the barriers and facilitators that influence engagement in physical activity among CRC patients. Although previous systematic reviews have examined the barriers and facilitators to exercise in cancer survivors [21], it is important to consider that colorectal cancer (CRC) patients may have distinct facilitators and barriers specifically related to the site of their disease. The purpose of this review is to comprehensively examine the literature and gain insights into the factors that facilitate, motivate, and hinder engagement in physical activity among colorectal cancer (CRC) patients.

## Methods

A scoping review was conducted to explore the existing literature on PA and exercise among CRC patients, following the PRISMA guidelines [22]. The scoping review is a rigorous method used to map research and present results in a format accessible to knowledge users. It is an increasingly common approach for mapping broad topics [23]. The six-step framework proposed by Arksey and O’Malley, which was further developed by Levec et al., comprises the following steps: (1) formulating the research question, (2) identifying relevant literature, (3) selecting studies, (4) charting the data, (5) synthesizing, summarizing, and reporting the data, and (6) consulting and translating knowledge. The review team consisted of knowledge users, along with a survivor of colorectal cancer (CRC), who actively participated in the consultation process to ensure the relevance of the findings to efforts related to PA and exercise provision and to facilitate the dissemination of the findings.

### Formulating the Research Question

Our research question was to explore the facilitators, preferences, and barriers to PA in CRC patients. For this review, PA was defined broadly to subsume lifestyle and leisure activity (i.e., any bodily movement produced by skeletal muscles that results in energy expenditure [24] as well as structured exercise (i.e., planned, structured, and repetitive and has as a final or an intermediate objective to improve or maintain physical fitness [24]. For this review, sport is defined as a recreational and/or competitive activity involving skill [24], also considered PA.

### Identifying Relevant Studies

The first author developed a comprehensive search strategy with expert research librarians (HY and ZR). The databases searched included PubMed, Web of Science, Embase, and Cochrane from their inception to 27 June 2023. The searches were exclusively conducted in English without any date or study type restrictions. Additionally, reference lists of the included articles were manually searched to identify any potentially eligible resources. Each title/abstract was screened by two authors independently based on the pre-determined selection criteria. Discrepancies were resolved through discussions. The same process was also applied to full-text review.

### Selecting Studies

The study selection criteria for this review are as follows:

#### Inclusion Criteria

a: Peer-reviewed published original research (randomized controlled trials, controlled studies, observational studies, qualitative studies, and mixed-methods studies). b: Studies reporting findings that address the research question about this population. c: Concerning CRC aged ≥18. Modification section: d: PA was defined broadly to subsume lifestyle and leisure activity (i.e., any bodily movement produced by skeletal muscles that results in energy expenditure [16]) as well as structured exercise (i.e., planned, structured, and repetitive and has as a final or an intermediate objective to improve or maintain physical fitness [16]). e: Specific cancer stages are not subject to special restrictions.

#### Exclusion Criteria

a: Editorials, commentaries, case studies, abstracts, and review articles.
b: Non-research articles (as this review only focused on research-related data).

### Charting the Data

Two authors abstracted the data using a standardized checklist, which two additional authors then checked. The data were categorized according to the research question and themes. The data abstracted included the first author, year, data type (i.e., quantitative, qualitative), study design (cross-sectional, cohort, randomized controlled trial, quasi-experimental), sample size, and participant characteristics (e.g., mean age) (Table 1). In addition to facilitators, motivators, preferences, and barriers, data reported as associations are also abstracted and coded as “correlates of PA.” Similar to the selection of studies, data extraction was carried out using a collaborative and iterative team approach [25]. Additionally, any disagreements were resolved by discussion.

**TABLE 1:** Study characteristics.

Study	Data type (related to this review)	Study design	Data collection method	Sample size	Age (mean age)	% Of comorbidities
Carla et al. (2019)	Qualitative	Cross-sectional	one-on-one interview	15	72.7	55%
Chloe et al. (2016)	Qualitative	Cross-sectional	Semi-structured interviews	24	69.38 ± 4.19	47%
Kerry et al. (2005)	Qualitative	Cross-sectional	Telephone interviews	69	59.9 ± 11.2	56%
Erin et al. (2012)	Quantitative	Cross-sectional	Mailed questionnaire	600	67.3	78%
Andria et al. (2016)	Quantitative	Prospective cohort	Telephone interviews	18	No report	55%
Kang et al. (2019)	Quantitative	Cross-sectional	questionnaire survey	168	61.26 ± 10.34	54%
Andrew et al. (2018)	Qualitative and Quantitative	Prospective cohort	questionnaire survey and Semi-structured interviews	44	No report	No report
Chou et al. (2016)	Quantitative	Cross-sectional	questionnaire survey	321	61.98 ± 11.45	No report
Lora et.al. (2015)	Quantitative	Cross-sectional	Mailed questionnaire	843	65.6 ± 11.7	No report
Brigid et al. (2009)	Quantitative	Cross-sectional	Telephone interviews	538	No report	36%
Abigail et al. (2016)	Quantitative	Cross-sectional	Mailed questionnaire	495	66.75 ± 10.86	45%

### Synthesizing, Summarizing, and Reporting Results

Two independent authors (HY and ZR) extracted all relevant themes. Subsequently, a thematic synthesis was conducted to categorize facilitators and barriers into common themes consistently mentioned or identified as important across multiple studies. In quantitative studies (surveys/questionnaires), the frequency of the barrier/facilitator was determined based on the number of times the response was provided (i.e., 50% of participants reported treatment-related side effects as a barrier to exercise). For qualitative studies (focus groups and interviews), frequent themes were determined by the study authors through thematic analysis of the material. Therefore, the themes more frequently observed in the literature were regarded as the prevailing barriers/facilitators to lifestyle change. A significant theme is defined as a theme that has been identified in a minimum of three individual studies.

## Results

### Study Selection

**FIGURE 1:**
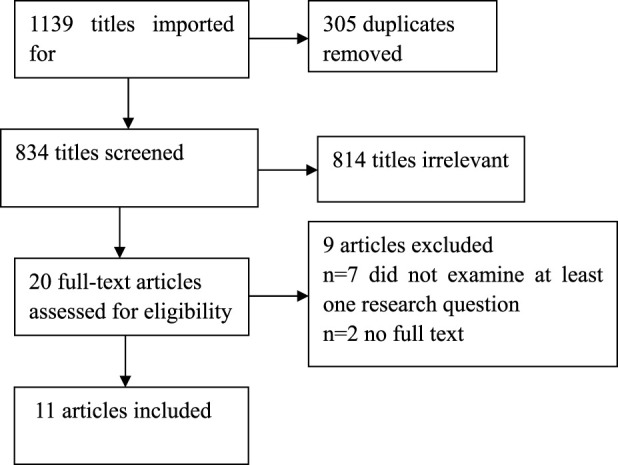
Illustrates the PRISMA flow diagram.

The search yielded 834 individual citations after removing duplicates. Following title and abstract screening, 20 underwent were selected for full-text review. Eleven articles that met the inclusion criteria were included in this scoping review. See Figure 1 for the PRISMA flowchart.

### Study Characteristics

Study characteristics of the 11 included studies are shown in Table 1. Seven studies reported quantitative data [1, 2, 26–30], three studies reported qualitative research approaches [31–33] and one study reported mixed methods [34]. The studies included in this analysis were published from 2005 to 2019 and were conducted in six different regions: The United Kingdom [1], Canada [2, 26, 27], the United States [29, 31, 34], the Netherlands [32], China [28, 30] and Australia [33].

### Overview of Findings

#### Purpose of Research and Study Designs

Most of the studies that reported quantitative data had a cross-sectional design (*n* = 5) [1, 2, 28–30], while others used an RCT or prospective research design (*n* = 2) [26, 27], which collected information relevant to this review as part of their report of their respective intervention studies.

### Obstacles and Facilitators to Physical Activity Implementation in Colorectal Cancer Patients

Modification section: This study indicates that the barriers and factors promoting PA in CRC patients are different from those in other types of cancer patients. The focus is on time constraints, the presence of stomas, and adverse weather conditions.

### Psychological Barriers

The “psychology” domains were identified as barriers and facilitators to physical activity. These factors include time constraints, aversion towards exercise, absence of motivation for physical activity, and the presence or absence of pleasure derived from exercising. The lack of time emerges as the most prominent barrier [1, 2, 26, 28, 31, 32], Modification section: Point out the most times. For example, I have to go to work, take care of my family after work, and engage in various social activities, so I don't have time for PA at all. Research reports have linked insufficient physical exercise to various time constraints, such as work obligations, family responsibilities, and social commitments [26, 28, 31]. One study emphasized that patients faced a significant obstacle in engaging in preoperative exercise due to the need to attend numerous hospital appointments, which posed a challenge to regular exercise [32]. Research reports indicate that individuals may lack motivation and enjoyment in physical activity due to their limited exercise skills or lack of interest in exercise [1, 26, 27, 31, 34].

### Environment Barriers

The “environment” domains were identified as barriers and facilitators to physical activity, including physical and social environments. The physical environment encompasses weather conditions. Three studies have indicated that poor weather is a significant barrier to physical activity [28, 31, 34]. One study reported that unsafe surroundings and a lack of appropriate facilities were also barriers to physical activity [26]. Social environment refers to support from the outside world. Research studies have reported that exercising can facilitate the formation of new friendships and motivate individuals to engage in physical activity [1, 31, 32]. Some participants emphasized the significance of peer support, while others indicated that the experiences of other patients did not apply to their situation or even hindered their engagement in the exercise program [32].

### Knowledge and Skills

The “knowledge” and “skills” domains were identified as barriers and facilitators to physical activity implementation. Several participants reported needing knowledge or skills to conduct physical activity assessments [31, 32]. According to the study reports, participants indicated a lack of familiarity with the recommended guidelines or perceived them as unsuitable for their situation [31, 32]. Some participants believed they already engaged in sufficient physical activity and did not perceive it as necessary [31, 32]. Lack of knowledge about physical activity from professionals is also a barrier to physical activity [31, 32].

### Disease Barriers

Nine studies have indicated that cancer, treatment side effects, and physical comorbidities hinder physical activity [1, 2, 26–30, 32, 34]. Modification section: (The included studies indicate that patients with physical comorbidities have significantly lower levels of physical activity.) Numerous studies have identified fatigue as the most prevalent disorder [1, 2, 26, 29, 30]. Due to the unique nature of the disease, research has shown that having a stoma is also a barrier for patients to engage in physical activities [26, 30].

### Perceived Benefits

Four studies highlighted the significance of recognizing and experiencing the benefits of physical activity as a critical factor in promoting physical activity [1, 27, 28, 32]. Participants indicated a lack of information regarding the significance of maintaining or enhancing their physical activity. Several participants reported experiencing physical and psychological benefits from engaging in physical activity, which facilitated their initiation of exercise despite their poor state of health.

## Discussion

Modification section: We found that CRC patients face a range of barriers to PA, some of which are similar to common barriers in patients and survivors of other cancer types, including absence of motivation for physical activity, lack of time, lack of PA knowledge, cancer, treatment side effects, and physical comorbidities [17]. However, due to differences in disease, CRC patients also have special barriers in following PA. For example, the presence of stomas.

In this scoping review, we provide an overview of the published literature that reported on facilitators, motivators, preferences, and barriers to engaging in PA and exercise programs among CRC. The findings of this review suggest that CRC may require special considerations in the planning and implementation of exercise programs and the promotion of physical activity due to the distinct characteristics of the disease. Specifically, the existence of a postoperative stoma not only induces shame among patients but also serves as a significant obstacle to engaging in physical activities. Modification section: Studies have shown [12] that after psychological intervention in colostomy patients, compared with the control group, the intervention group showed a significant improvement in negative emotions such as anxiety and depression, and was able to adapt to the stoma as soon as possible and engage in regular physical activity. Therefore, providing psychological care and encouraging patients to join stoma associations is crucial as they can significantly aid in addressing psychological issues.

Time constraints and fatigue pose a common barrier to PA for CRC Patients. Consequently, high-intensity interval training (HIIT), which involves repeated bouts of short duration, high-intensity PA intermingled with periods of lower-intensity active recovery, has emerged as a potential solution. Previous studies have investigated the use of HIIT in other cancer survivors [35, 36]. The results have demonstrated enhanced cardiovascular fitness in patients and improved cardiac regulation and stress responses in specific individuals [35]. Shorter-duration programs can reduce time constraints related to moderate-intensity interventions. Moreover, offering guidance on integrating physical activity into daily routines can encourage individuals to engage in physical activity, such as taking the stairs, walking to a destination instead of driving, taking a quick walk, exercising while watching TV, visiting nearby friends instead of calling them etc.

Improving health and engaging in social interaction are frequently cited as key motivations for exercise, according to the studies analyzed in this review. These findings are consistent with those observed in patients diagnosed with other types of cancer [37]. Family support is crucial as a source of social support for CRC patients. Participants reported a preference for exercising individually at home. Although group exercise provided an opportunity to interact with other patients, patients were reluctant to discuss their cancer diagnosis with others in the group [32]. Hence, further research is necessary to investigate the adoption and compliance with home-based exercise programs, as well as to determine the possibility of attaining exercise intensities that are adequately high and progressive to enhance the physical capacity of patients.

The significance of providing exercise advice and guidance for PA is apparent. However, research indicates that more than half of the participants still need to receive specific exercise advice or guidance. The advice was limited to general recommendations to increase PA for those who did. Healthcare professionals play a dual role as barriers and facilitators in promoting physical activity engagement among CRC patients. The study revealed that professionals lacked confidence in the advice provided. In particular, patients may need more confidence in participating in physical activity due to a lack of confidence or consistent advice from healthcare providers. Hence, training healthcare professionals on the advantages of guiding CRC patients on physical activity during their appointments could be beneficial. Moreover, participants suggested that customizing the advice to each patient and adapting it according to their changing needs enhances their adherence to the recommendations.

Our study revealed that CRC patients face various barriers to PA and exercise engagement. Some of these barriers are consistent with those commonly observed in patients and survivors of different cancer types, including cancer treatment side effects, comorbidities and fatigue [38]. The findings of this review also suggest the importance of considering not only the known challenges related to individual behavioral change and environmental barriers but also issues specific to CRC and its treatments. Fecal incontinence and diarrhea are frequently reported symptoms related to cancer and its treatment in patients with CRC [2, 26, 27, 32]. Reducing the intensity and duration of exercise can help address issues with incontinence. Engaging in physical activity at locations with nearby washrooms, such as shopping malls or community parks, is also beneficial [39]. Paradoxically, research suggests exercise can improve fecal incontinence and diarrhea in CRC patients during and after treatment. However, it is worth noting that these conditions can also be barriers to engaging in physical activity [40, 41].

Although adherence to PA or exercise is not commonly explored in studies, the factors influencing initiation and participation can also affect adherence. However, older adults with cancer can experience a significant decline in functional abilities, and patients with CRC also encounter distinct challenges associated with functional decline [42]. Given that over 70% of those diagnosed with CRC are aged 65 and above [32], it is reasonable to expect that patients initiating exercise programs may encounter difficulties maintaining their regimen, whether informal physical activity or structured programs. Lastly, while our primary focus was not to analyze the delivery format of exercise programs, we observed that most existing studies on physical activity and exercise programs predominantly involve site-based group exercise interventions or unsupervised home-based independent exercise activities. To address this issue, it is essential to explore an alternative approach where exercise interventions are provided through virtual platforms. These interventions should be conducted in a group-based, live, supervised format, allowing patients to participate safely and conveniently in their own homes.

### Limitations and Strengths

One of the strengths of this review is the implementation of an extensive search strategy, which successfully identified 11 articles that satisfied the predefined eligibility criteria. Most studies produced consistent findings, although the emphasis varied depending on the participants involved. This comprehensive review does not impose any methodological restrictions on the included studies. Therefore, studies that met the inclusion criteria included questionnaires/surveys and semi-structured interviews. Due to inherent differences, particularly between questionnaires/surveys and interviews, participants may exhibit significant differences in their responses to specific questions and their reportage of barriers and facilitators of PA. Additionally, the selection of reporting personal barriers and facilitators may differ between questionnaires and interviews. For instance, a questionnaire may ask directly about age as a possible obstacle to making lifestyle changes, while an interview might pose guiding questions that tackle age-related barriers more indirectly. Therefore, this could influence the participants’ responses and the subsequent categorization of major themes. Given the limited literature in this field, all study designs are equally included in the analysis. Hence, it is challenging to accurately ascertain the prevalence of major themes and their respective significance about each other due to the potential impact of research design on the reporting of barriers and facilitators.

This review obtained feedback from 3135 CRC patients across 11 studies. These individuals varied in age, population, treatments, and duration of treatment. However, the research was limited to six regions: the United Kingdom, Canada, the United States, the Netherlands, China and Australia. Although we identified similar barriers and facilitators for PA changes among these populations, it is essential to exercise caution when applying these findings to similar populations in other regions.

### Implications

Physical activity is associated with reduced overall mortality and colorectal cancer (CRC) mortality. The research revealed that engaging in sports activities such as golf, bowling, and curling for a longer duration every week was associated with a lower risk of colorectal cancer recurrence and improved disease and survival outcomes [15, 18]. In addition, exercises and programs with an aerobic component have been shown to improve cardiovascular endurance, self-esteem, and quality of life in CRC [27]. Importantly, aerobic exercise is the most common form of treatment for CRC. Aerobic exercise therapy is administered to nearly 50% of patients diagnosed with CRC. Adverse side effects commonly include fatigue, diarrhea, skin irritation, and hand-foot syndrome [27]. Exercise has been shown to benefit patients in preventing or reversing treatment-related side effects [40, 43–47]. Therefore, it is essential to initiate a discussion concerning exercise and appropriate referrals to alleviate the adverse effects of aerobic exercise.

From a research perspective, considering the unique needs of CRC, patient-engaged research can be advantageous in designing research studies and exercise interventions. This approach focuses on tailoring and accommodating individual preferences and needs while ensuring feasibility and acceptability among the population. Furthermore, additional research is necessary to investigate the factors associated with exercise adherence in this population, considering the difficulties and barriers related to treatment-side effects and functional declines. Moreover, researching to identify strategies that can help clinicians overcome barriers to initiating exercise-related discussions with patients may also be beneficial. Furthermore, it is necessary to extend the scope to include investigating online exercise programs that are group-based, supervised, and conducted remotely from home. This research should focus on assessing the feasibility and efficacy and consider aspects of acceptability, preferences, and potential barriers.

In clinical practice, oncology clinicians should regularly assess patients’ current level of physical activity, initiate discussions about the benefits of physical activity and exercise, advise patients to maintain an active lifestyle and refer patients (unless contraindicated) to appropriate programs. Modification section: However, these may vary depending on the medical environment or available resources. Whenever possible, patients should be referred to cancer exercise specialists or other qualified healthcare professionals for further evaluation and exercise recommendations and prescriptions [48]. Regarding the timing of introducing the subject of physician assistance (PA) and illustrating its potential benefits within the context of patient care (PC), it is advisable to address it sooner rather than later. Ideally, this should occur before the initiation of treatment or even during the watch-and-wait phase (a potentially calmer time for the patient), gastroenterologist and/or family physicians.

## Conclusion

CRC patients have unique facilitators and barriers concerning PA and exercise program participation. Further efforts are required from both the research and clinical practice perspectives to facilitate the participation and adherence of this population in regular physical activity and exercise programs. Modification section: In the future, a prospective multicenter study may also be proposed to explore the impact of PA on the quality of life of CRC patients.

## Data Availability

The original contributions presented in the study are included in the article/Supplementary material, further inquiries can be directed to the corresponding author.

## References

[1] Fisher A Wardle J Beeken RJ Croker H Williams K Grimmett C . Perceived Barriers and Benefits to Physical Activity in Colorectal Cancer Patients. Support Care Cancer (2016) 24:903–10. 10.1007/s00520-015-2860-0 26268781 PMC4689774

[2] McGowan EL Speed-Andrews AE Rhodes RE Blanchard CM Culos-Reed SN Friedenreich CM Sport Participation in Colorectal Cancer Survivors: An Unexplored Approach to Promoting Physical Activity. Support Care Cancer (2013) 21:139–47. 10.1007/s00520-012-1501-0 22639138

[3] Courneya KS Katzmarzyk PT Bacon E . Physical Activity and Obesity in Canadian Cancer Survivors: Population-Based Estimates From the 2005 Canadian Community Health Survey. Cancer (2008) 112:2475–82. 10.1002/cncr.23455 18428195

[4] van Waart H van Harten WH Buffart LM Sonke GS Stuiver MM Aaronson NK . Why Do Patients Choose (Not) to Participate in an Exercise Trial During Adjuvant Chemotherapy for Breast Cancer? Psychooncology (2016) 25:964–70. 10.1002/pon.3936 26282696

[5] Carli F Scheede-Bergdahl C . Prehabilitation to Enhance Perioperative Care. Anesthesiology Clin (2015) 33:17–33. 10.1016/j.anclin.2014.11.002 25701926

[6] Grimmett C Bridgewater J Steptoe A Wardle J . Lifestyle and Quality of Life in Colorectal Cancer Survivors. Qual Life Res (2011) 20:1237–45. 10.1007/s11136-011-9855-1 21286822

[7] Lynch BM Cerin E Newman B Owen N . Physical Activity, Activity Change, and Their Correlates in a Population-Based Sample of Colorectal Cancer Survivors. Ann Behav Med (2007) 34:135–43. 10.1007/bf02872668 17927552

[8] Fisher A Smith L Wardle J . Physical Activity Advice Could Become Part of Routine Care for Colorectal Cancer Survivors. Future Oncol (2016) 12:139–41. 10.2217/fon.15.269 26616343

[9] Lynch BM Cerin E Owen N Hawkes AL Aitken JF . Prospective Relationships of Physical Activity With Quality of Life Among Colorectal Cancer Survivors. J Clin Oncol (2008) 26:4480–7. 10.1200/jco.2007.15.7917 18802160

[10] Peddle CJ Au HJ Courneya KS . Associations Between Exercise, Quality of Life, and Fatigue in Colorectal Cancer Survivors. Dis Colon Rectum (2008) 51:1242–8. 10.1007/s10350-008-9324-2 18536970

[11] Mayo NE Feldman L Scott S Zavorsky G Kim DJ Charlebois P Impact of Preoperative Change in Physical Function on Postoperative Recovery: Argument Supporting Prehabilitation for Colorectal Surgery. Surgery (2011) 150:505–14. 10.1016/j.surg.2011.07.045 21878237

[12] Santa Mina D Clarke H Ritvo P Leung YW Matthew AG Katz J Effect of Total-Body Prehabilitation on Postoperative Outcomes: A Systematic Review and Meta-Analysis. Physiotherapy (2014) 100:196–207. 10.1016/j.physio.2013.08.008 24439570

[13] Huang GH Ismail H Murnane A Kim P Riedel B . Structured Exercise Program Prior to Major Cancer Surgery Improves Cardiopulmonary Fitness: A Retrospective Cohort Study. Support Care Cancer (2016) 24:2277–85. 10.1007/s00520-015-3028-7 26590843

[14] Holmes MD Chen WY Feskanich D Kroenke CH Colditz GA . Physical Activity and Survival After Breast Cancer Diagnosis. JAMA (2005) 293:2479–86. 10.1001/jama.293.20.2479 15914748

[15] Meyerhardt JA Giovannucci EL Holmes MD Chan AT Chan JA Colditz GA Physical Activity and Survival After Colorectal Cancer Diagnosis. J Clin Oncol (2006) 24:3527–34. 10.1200/jco.2006.06.0855 16822844

[16] Meyerhardt JA Heseltine D Niedzwiecki D Hollis D Saltz LB Mayer RJ Impact of Physical Activity on Cancer Recurrence and Survival in Patients With Stage III Colon Cancer: Findings From CALGB 89803. J Clin Oncol (2006) 24:3535–41. 10.1200/jco.2006.06.0863 16822843

[17] Holick CN Newcomb PA Trentham-Dietz A Titus-Ernstoff L Bersch AJ Stampfer MJ Physical Activity and Survival After Diagnosis of Invasive Breast Cancer. Cancer Epidemiol Biomarkers Prev (2008) 17:379–86. 10.1158/1055-9965.epi-07-0771 18250341

[18] Meyerhardt JA Giovannucci EL Ogino S Kirkner GJ Chan AT Willett W Physical Activity and Male Colorectal Cancer Survival. Arch Intern Med (2009) 169:2102–8. 10.1001/archinternmed.2009.412 20008694 PMC2852183

[19] Je Y Jeon JY Giovannucci EL Meyerhardt JA . Association Between Physical Activity and Mortality in Colorectal Cancer: A Meta-Analysis of Prospective Cohort Studies. Int J Cancer (2013) 133:1905–13. 10.1002/ijc.28208 23580314

[20] Courneya KS Friedenreich CM . Relationship Between Exercise Pattern Across the Cancer Experience and Current Quality of Life in Colorectal Cancer Survivors. J Altern Complement Med (1997) 3:215–26. 10.1089/acm.1997.3.215 9430325

[21] Clifford BK Mizrahi D Sandler CX Barry BK Simar D Wakefield CE Barriers and Facilitators of Exercise Experienced by Cancer Survivors: A Mixed Methods Systematic Review. Support Care Cancer (2018) 26:685–700. 10.1007/s00520-017-3964-5 29185105

[22] Moher D Liberati A Tetzlaff J Altman DG . Preferred Reporting Items for Systematic Reviews and Meta-Analyses: The PRISMA Statement. Plos Med (2009) 6:e1000097. 10.1371/journal.pmed.1000097 19621072 PMC2707599

[23] Pham MT Rajic A Greig JD Sargeant JM Papadopoulos A McEwen SA . A Scoping Review of Scoping Reviews: Advancing the Approach and Enhancing the Consistency. Res Synth Methods (2014) 5:371–85. 10.1002/jrsm.1123 26052958 PMC4491356

[24] Caspersen CJ Powell KE Christenson GM . Physical Activity, Exercise, and Physical Fitness: Definitions and Distinctions for Health-Related Research. Public Health Rep (1985) 100:126–31. 3920711 PMC1424733

[25] Levac D Colquhoun H O'Brien KK . Scoping Studies: Advancing the Methodology. Implementation Sci (2010) 5:69. 10.1186/1748-5908-5-69 PMC2954944 20854677

[26] Courneya KS Friedenreich CM Quinney HA Fields AL Jones LW Vallance JK A Longitudinal Study of Exercise Barriers in Colorectal Cancer Survivors Participating in a Randomized Controlled Trial. Ann Behav Med (2005) 29:147–53. 10.1207/s15324796abm2902_9 15823788

[27] Morielli AR Usmani N Boule NG Severin D Tankel K Nijjar T Exercise Motivation in Rectal Cancer Patients During and After Neoadjuvant Chemoradiotherapy. Support Care Cancer (2016) 24:2919–26. 10.1007/s00520-016-3110-9 26847350

[28] Chou YJ Lai YH Lin BR Liang JT Shun SC . Factors Influencing Amount of Weekly Exercise Time in Colorectal Cancer Survivors. Cancer Nurs (2017) 40:201–8. 10.1097/ncc.0000000000000383 27135754

[29] Packel LB Prehn AW Anderson CL Fisher PL . Factors Influencing Physical Activity Behaviors in Colorectal Cancer Survivors. Am J Health Promot (2015) 30:85–92. 10.4278/ajhp.140103-quan-7 25372238

[30] Kang DQ Li Y Chen ZQ Liu Q Su CX Guo H Correlates of Physical Activity in Colorectal Cancer Patients Based on Health Promotion Model. Cancer Nurs (2020) 43:E264–72. 10.1097/ncc.0000000000000725 32813487

[31] Maxwell-Smith C Zeps N Hagger MS Platell C Hardcastle SJ . Barriers to Physical Activity Participation in Colorectal Cancer Survivors at High Risk of Cardiovascular Disease. Psychooncology (2017) 26:808–14. 10.1002/pon.4234 27478009

[32] Agasi-Idenburg CS Zuilen MK Westerman MJ Punt C Aaronson NK Stuiver MM . I Am Busy Surviving" - Views About Physical Exercise in Older Adults Scheduled for Colorectal Cancer Surgery. J Geriatr Oncol (2020) 11:444–50. 10.1016/j.jgo.2019.05.001 31122871

[33] Lynch BM Owen N Hawkes AL Aitken JF . Perceived Barriers to Physical Activity for Colorectal Cancer Survivors. Support Care Cancer (2010) 18:729–34. 10.1007/s00520-009-0705-4 19636595

[34] Ray AD Masucci Twarozek A Williams BT Erwin DO Underwood WR Mahoney MC . Exercise in African American and White Colorectal Cancer Survivors: A Mixed Methods Approach. Rehabil Oncol (2018) 36:188–97. 10.1097/01.reo.0000000000000125 30467528 PMC6241321

[35] Toohey K Pumpa K McKune A Cooke J Welvaert M Northey J The Impact of High-Intensity Interval Training Exercise on Breast Cancer Survivors: A Pilot Study to Explore Fitness, Cardiac Regulation and Biomarkers of the Stress Systems. Bmc Cancer (2020) 20:787. 10.1186/s12885-020-07295-1 32819304 PMC7441660

[36] Adams SC DeLorey DS Davenport MH Stickland MK Fairey AS North S Effects of High-Intensity Aerobic Interval Training on Cardiovascular Disease Risk in Testicular Cancer Survivors: A Phase 2 Randomized Controlled Trial. Cancer (2017) 123:4057–65. 10.1002/cncr.30859 28708930

[37] Brunet J Taran S Burke S Sabiston CM . A Qualitative Exploration of Barriers and Motivators to Physical Activity Participation in Women Treated for Breast Cancer. Disabil Rehabil (2013) 35:2038–45. 10.3109/09638288.2013.802378 23772995

[38] Granger CL Connolly B Denehy L Hart N Antippa P Lin KY Understanding Factors Influencing Physical Activity and Exercise in Lung Cancer: A Systematic Review. Support Care Cancer (2017) 25:983–99. 10.1007/s00520-016-3484-8 27900549

[39] Wildes TM Fiala MA . Falls in Older Adults With Multiple Myeloma. Eur J Haematol (2018) 100:273–8. 10.1111/ejh.13009 29239009 PMC5814335

[40] Taaffe DR Newton RU Spry N Joseph D Chambers SK Gardiner RA Effects of Different Exercise Modalities on Fatigue in Prostate Cancer Patients Undergoing Androgen Deprivation Therapy: A Year-Long Randomised Controlled Trial. Eur Urol (2017) 72:293–9. 10.1016/j.eururo.2017.02.019 28249801

[41] Horgan S O'Donovan A . The Impact of Exercise During Radiation Therapy for Prostate Cancer on Fatigue and Quality of Life: A Systematic Review and Meta-Analysis. J Med Imaging Radiat Sci (2018) 49:207–19. 10.1016/j.jmir.2018.02.056 32074040

[42] Resnick MJ Koyama T Fan KH Albertsen PC Goodman M Hamilton AS Long-Term Functional Outcomes After Treatment for Localized Prostate Cancer. N Engl J Med (2013) 368:436–45. 10.1056/nejmoa1209978 23363497 PMC3742365

[43] Lam T Cheema B Hayden A Lord SR Gurney H Gounden S Androgen Deprivation in Prostate Cancer: Benefits of Home-Based Resistance Training. Sports Med Open (2020) 6:59. 10.1186/s40798-020-00288-1 33315154 PMC7736381

[44] Teleni L Chan RJ Chan A Isenring EA Vela I Inder WJ Exercise Improves Quality of Life in Androgen Deprivation Therapy-Treated Prostate Cancer: Systematic Review of Randomised Controlled Trials. Endocrine-Related Cancer (2016) 23:101–12. 10.1530/erc-15-0456 26584972

[45] Gardner JR Livingston PM Fraser SF . Effects of Exercise on Treatment-Related Adverse Effects for Patients With Prostate Cancer Receiving Androgen-Deprivation Therapy: A Systematic Review. J Clin Oncol (2014) 32:335–46. 10.1200/jco.2013.49.5523 24344218

[46] Cheung AS Zajac JD Grossmann M . Muscle and Bone Effects of Androgen Deprivation Therapy: Current and Emerging Therapies. Endocr Relat Cancer (2014) 21:R371–94. 10.1530/erc-14-0172 25056176

[47] Sattar S Haase KR Bradley C Papadopoulos E Kuster S Santa Mina D Barriers and Facilitators Related to Undertaking Physical Activities Among Men With Prostate Cancer: A Scoping Review. Prostate Cancer Prostatic Dis (2021) 24:1007–27. 10.1038/s41391-021-00399-0 34108646

[48] Schmitz KH Campbell AM Stuiver MM Pinto BM Schwartz AL Morris GS Exercise Is Medicine in Oncology: Engaging Clinicians to Help Patients Move Through Cancer. CA: A Cancer J Clinicians (2019) 69:468–84. 10.3322/caac.21579 PMC7896280 31617590

